# Histological Features of Sporadic and Familial Testicular Germ Cell Tumors Compared and Analysis of Age-Related Changes of Histology

**DOI:** 10.3390/cancers13071652

**Published:** 2021-04-01

**Authors:** Andreas Stang, Mary L. McMaster, Isabell A. Sesterhenn, Elizabeth Rapley, Robert Huddart, Ketil Heimdal, Katherine A. McGlynn, Jan Wolter Oosterhuis, Mark H. Greene

**Affiliations:** 1 Institut für Medizinische Informatik, Biometrie und Epidemiologie, Universitätsklinikum Essen, 45147 Essen, Germany; imibe.dir@uk-essen.de; 2 School of Public Health, Department of Epidemiology, Boston University, Boston, MA 02118, USA; 3 Clinical Genetics Branch, Division of Cancer Epidemiology and Genetics, National Cancer Institute, National Institutes of Health, Rockville, MD 20850, USA; greenem@mail.nih.gov; 4 Joint Pathology Center, Silver Spring, MD 20910, USA; isabell.a.sesterhenn.civ@mail.mil; 5 Division of Genetics and Epidemiology, Institute for Cancer Research, London SM7 1DN, UK; liz@madebyedge.com; 6 Division of Radiotherapy and Imaging, Institute for Cancer Research, London SM7 1DN, UK; Robert.Huddart@icr.ac.uk; 7 Department of Medical Genetics, Oslo University Hospital Rikshospitalet, 0027 Oslo, Norway; kheimdal@ous-hf.no; 8 Metabolic Epidemiology Branch, Division of Cancer Epidemiology and Genetics, National Cancer Institute, National Institutes of Health, Rockville, MD 20850, USA; mcglynnk@mail.nih.gov; 9 Department of Pathology, Josephine Nefkens Institute, Erasmus University Medical Center, 3000 DR Rotterdam, The Netherlands; j.w.oosterhuis@erasmusmc.nl

**Keywords:** neoplasm, germ cell and embryonal, testicular microlithiasis, registries, histology, classification, age distribution, pathogenesis, advances in pathological assessment of GCT

## Abstract

**Simple Summary:**

Testicular germ cell tumors (TGCT) are highly heritable, and earlier studies reported a higher prevalence of certain microscopic features in familial cases compared with sporadic cases. Reasoning by analogy relative to different causal genes for different histologic subtypes of familial kidney cancer, we searched for etiologically informative histopathology associations in familial testicular germ cell cancer. We conducted a detailed, blinded pathology review of familial and sporadic TGCT cases to investigate whether we could identify differences between these two patient subsets and to study the effect of age at diagnosis on histologic features in both groups combined. Our results show no specific histologic differences between familial and sporadic TGCTs. However, we observed histologic features that varied with age at diagnosis among the two groups combined. Thus, our results suggest that there are no histological differences between familial and sporadic TGCT that might identify genetically distinct disease subsets.

**Abstract:**

This study aimed to compare histological features of familial and sporadic testicular germ cell tumors (TGCTs) and surrounding parenchyma, since discriminating features might be etiologically relevant and clinically useful. The study of parenchyma was prompted by reports claiming a higher prevalence of testicular microlithiasis in familial cases. Histological features of TGCTs and surrounding parenchyma of 296 sporadic and 305 familial cases were compared. For each case, one representative hematoxylin and eosin-stained slide was available. Slides were independently scored by two expert pathologists using a semi-quantitative data abstract. Discrepancies were resolved by consensus. A logistic regression model was used to assess the ability to discriminate between sporadic and familial GCT. The histological composition of a tumor, amount of lymphocytic infiltration, amount of germ cell neoplasia in situ (GCNIS), and presence of testicular microlithiasis (TM) did not discriminate between sporadic and familial GCT (area under the curve 0.56, 95%CI 0.51–0.61). Novel observations included increasing lymphocytic infiltration and decreasing GCNIS and TM with increasing age at diagnosis. The presence of tubules with infiltrating lymphocytes was mainly associated with pure seminomas and nonseminomas with a seminoma component. Among seminomas, tubules with infiltrating lymphocytes decreased with increasing age. No discernable differences between sporadic and familial TGCTs were found. The age-related changes in the tumors and surrounding parenchyma in these groups combined are consistent with a host response building up over time predominantly affecting seminomas, the seminoma-component of nonseminomas and GCNIS. TM may gradually dissolve with age. Our hypothesis that histological differences between sporadic and familial TGCT might identify genetically distinct disease subsets was not supported.

## 1. Introduction

Testicular germ cell tumors (TGCT) are the most commonly occurring cancers among men aged 15–20 years in many countries. Incidence rates are highest in northern European countries but have been increasing at a more rapid rate in lower incidence countries [1]. Reasons for the increasing rates are unclear although there are several well-identified risk factors, including cryptorchidism [2], family history [3,4], subfertility [5], and contralateral testicular cancer [6]. It is known, however, that TGCT has one of the highest heritabilities of any cancer [3]. As such, men who have an affected first-degree relative are at marked increased risk of TGCT. Whether the tumors of familial and sporadic cases differ in histologic distribution or in features of the tumors however, is not clear. Attempts to identify highly penetrant cancer susceptibility genes have been unsuccessful [7] and large-scale sequencing of TGCT cases has excluded a major predisposition gene [8]. Currently, this heritability is considered polygenic, i.e., the combined effects of multiple common risk alleles, each of small effect [9].

This study concerns post-pubertal TGCTs: seminoma and nonseminoma, of which germ cell neoplasia in situ (GCNIS) is the common precursor lesion (type II GCTs). The default progression of GCNIS is towards seminoma; nonseminoma arises when an intratubular or invasive seminomatous cell is reprogrammed to an embryonal carcinoma (EC) cell, the totipotent stem cell of nonseminoma, which gives rise to the other nonseminomatous components [10].

An inflammatory infiltrate with a prominent lymphocytic component is an integral part of seminoma histology. This host response to seminoma also seems to affect GCNIS and intratubular seminoma in the surrounding parenchyma, in view of lymphocytes surrounding and infiltrating into involved tubules [11].

Nonseminoma also usually has an inflammatory reaction, which not so obviously affects tubules with GCNIS and intratubular seminoma in the parenchyma surrounding the tumor. Microliths of about 1–2 mm in diameter, composed of concentric layers of hydroxyapatite, most often localized in tubules affected by GCNIS, comprise a conspicuous feature of parenchyma adjacent to TGCTs. Microliths are visible on ultrasound scans of the testis and may raise suspicion of a TGCT in men at risk. Reportedly, microlithiasis is more frequent in familial than sporadic cases of TGCT [12]. The well-known histologic complexity of TGCT suggested the possibility that pathology-defined subsets of familial cases, if they existed, might permit more efficient detection of genetic etiology in one or more subgroups, in a manner similar to that observed in hereditary kidney cancers [13,14].

The aim of this study was to investigate whether sporadic and familial cases of TGCTs differed in terms of histological features of the tumor and its surrounding parenchyma. Furthermore, we wanted to examine the effect of age at diagnosis on several histopathological features including GCNIS, testicular microlithiasis (TM), and lymphocytic infiltration, as well as seminiferous tubules with infiltrating lymphocytes.

## 2. Materials and Methods

### 2.1. Sampling of Familial and Sporadic Testicular Germ Cell Tumors

Familial cases of TGCT (FTGCT) were ascertained and enrolled by selected members of the International Testicular Cancer Linkage Consortium (ITCLC) [15]. Each institution contributed all its familial cases that had pathology material available. FTGCT cases were defined as the presence among genetically related men of at least two cases of documented TGCT. The cases included seminoma (International Classification of Diseases for Oncology, 3rd edition [16], ICD-O code 9061/3), embryonal carcinoma (9070/3), endodermal sinus (yolk sac) tumor (9071/3), gonadoblastoma (9073/1), choriocarcinoma (9100/3), teratoma–postpubertal type (9080/3) or teratoma with somatic-type malignancy (9084/3), and mixed germ cell tumors (9085/3).

In addition to the familial TGCT cases, each participating pathology center (U.S. National Cancer Institute, UK Institute of Cancer Research, and the Institute for Cancer Research, Department of Genetics and Department of Oncology, The Norwegian Radium Hospital) sampled sporadic TGCTs from its pathology archive. From the pathology accession records, after a familial TGCT case was chosen, the next non-familial TGCT case accessioned. The planned ratio of familial to sporadic cases was 1:1, with a planned study size of 440 TGCTs. At each center, pathology reports and one representative histological HE-stained slide were retrieved.

### 2.2. Central Pathology Review

The central pathology review was based on one representative histological slide (H and E stained). The slide was assigned a study ID and all other identifying information was expunged. Thereafter, a central pathology review board consisting of IAS and JWO reviewed the slides independently in a blinded fashion. For all cases, the histological features of the tumors and characteristics of the adjacent parenchyma were scored using a pathology review form in agreement with the updated WHO classification [17]. Discrepancies between the pathologists were resolved by consensus. Subsequently, TGCTs were grouped into three groups: nonseminomas (with one or more nonseminomatous components), tumors combining seminoma with one or more nonseminomatous components, and pure seminomas.

GCNIS, as first described by Skakkebaek et al. in 1972 [18], was diagnosed in agreement with the updated WHO classification [17]. As GCNIS is a multifocal and polyclonal process [19], a scoring (none, occasional, some, many) was applied based on the number of seminiferous tubules involved in the slides, which could be due either to a unifocal or multifocal process.

**Figure 1:**
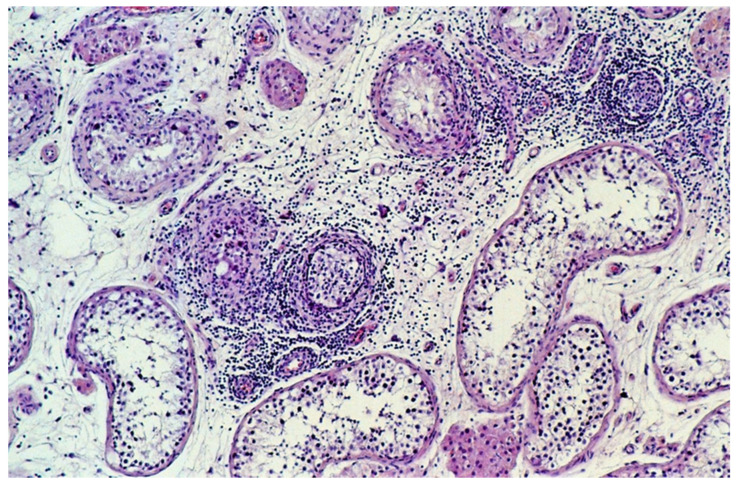
Tubules with infiltrating lymphocytes. The lymphocytic host response targets tubules with GCNIS, not the tubules with spermatogenesis. The diameter of the tubules decreases as GCNIS is gradually cleared. (hematoxylin and eosin staining, ×100 magnification).

The extent of infiltrating lymphocytes within the tumor and surrounding parenchyma was classified as none, slight, moderate, or extensive. Tubules with infiltrating lymphocytes were separately scored, whereby a single lymphocyte in the wall or the lumen of a seminiferous tubule resulted in its being designated a tubule with infiltrating lymphocytes (Figure 1) and categorized on an ordinal scale as none, occasional (1–5), some (6–20), or many (>20).

TM was defined as the presence of round calcifications, often with concentric layers, most often located within the tubules. Microliths were coded as absent, present or unable-to-evaluate.

### 2.3. Statistical Methods

A logistic regression model was used to assess the ability to discriminate between sporadic and familial TGCT. The following histological variables were included in the analysis: amount of lymphocytic infiltration, GCNIS (both as class variables), tubules with infiltrating lymphocytes, presence of TM, and percentage of each histological element. In addition, the area under the receiver operating curve (AUROC) was estimated and assessed via calibration by the Hosmer-Lemeshow test [20]. To estimate prevalence differences between histological groups, we used linear regression. We calculated 95% confidence intervals (95%CI) to assess the precision of our estimates because our goal was estimation rather than significance testing [21,22].

To examine the effect of age at diagnosis on histopathological features, the data of the familial and sporadic cases were combined.

## 3. Results

Overall, the study included 601 TGCTs; 296 were sporadic and 305 were familial. The age distribution among sporadic and familial GCT cases was very similar, with a mean age of 33.2 years (standard deviation [SD] 9.8). The prevalence of seminoma, seminoma plus nonseminoma, and nonseminoma was similar between groups, with a slightly higher percentage of seminoma among familial than sporadic TGCTs (Table 1).

The frequency of GCNIS, TM, and lymphocytic infiltration was very similar between the sporadic and familial TGCTs (Table 2). Stratification by histological group, although based on smaller numbers, revealed that familial pure nonseminoma (prevalence difference: 16.3, 95%CI −5.8; 38.4) and seminoma plus nonseminoma (prevalence difference: 10.8, 95%CI −11.1; 32.8) had a higher prevalence of TM than did the sporadic cases (Table S1). Adjustment for age at diagnosis did not markedly change these prevalence differences.

A multiple logistic regression model that included the percentages of histological components present in a tumor, amount of lymphocytic infiltration, amount of GCNIS and presence of TM, assessed the ability to discriminate between sporadic and familial TGCT. The estimated area under the curve (AUC) was 0.56 (95%CI 0.51–0.61), indicating little to no discrimination (Figure S1).

**Figure 2:**
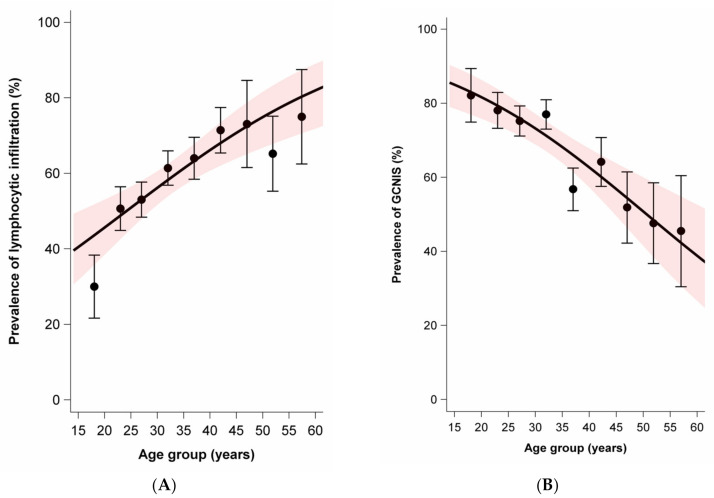

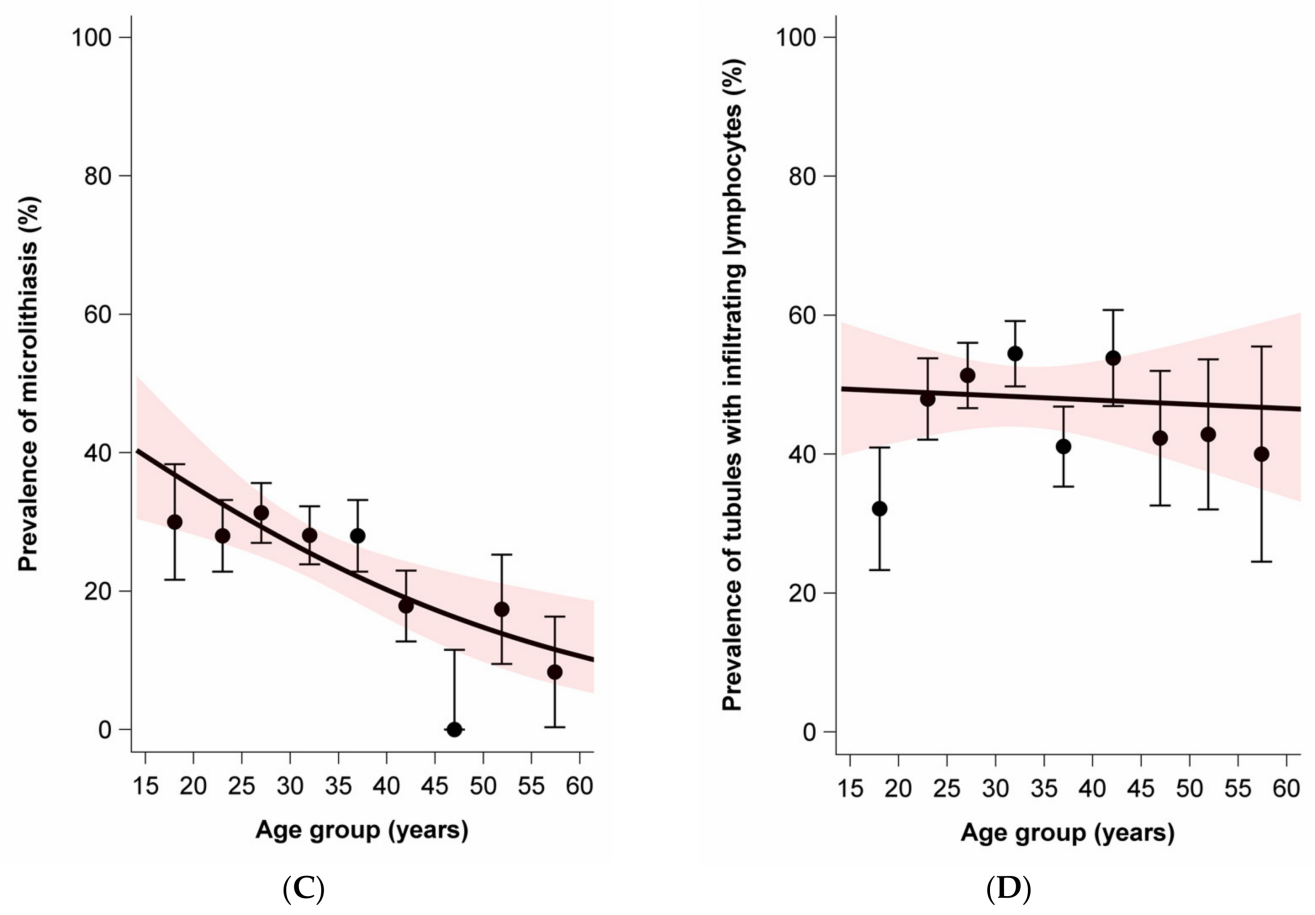
Age dependence of lymphocytic infiltration, germ cell neoplasia in situ (GCNIS), testicular microlithiasis, and tubules with infiltrating lymphocytes among sporadic and familial testicular germ cell tumors. Dots and whiskers indicate the age group specific prevalence +/− 1 standard error; the red bands display the 95% confidence interval (95% CI) bands; all models include age only; odds ratios (OR) per 5-year age increase. (**A**) Prevalence of lymphocytic infiltration by age at diagnosis. OR = 1.21 (95% CI: 1.11–1.33). (**B**) Prevalence of GCNIS by age at diagnosis. OR = 0.78 (95%CI: 0.71–0.87). (**C**) Prevalence of microlithiasis by age at diagnosis. OR = 0.83 (95%CI: 0.74–0.93). (**D**) Prevalence of tubules with infiltrating lymphocytes. OR = 0.99 (95%CI: 0.90–1.08).

While the average age was slightly lower for nonseminoma (mean 28.8, SD 9.0) than seminoma plus nonseminoma (mean 31.2, SD 7.4), the average age was notably higher for seminoma (mean 37.0, SD 9.5). Age at diagnosis was positively associated with moderate to extensive lymphocytic infiltration in tumors and parenchyma. In contrast, age at diagnosis was negatively associated with the presence of GCNIS and the presence of TM. There was no association between age at diagnosis and the prevalence of tubules with infiltrating lymphocytes (Figure 2).

The presence of GCNIS was not clearly associated with age for pure nonseminona. However, among seminoma-plus-nonseminoma and pure seminoma, the presence of some-to-many GCNIS was negatively associated with age. The higher the age, the less frequent was the presence of GCNIS (Figure S2).

**Figure 3:**
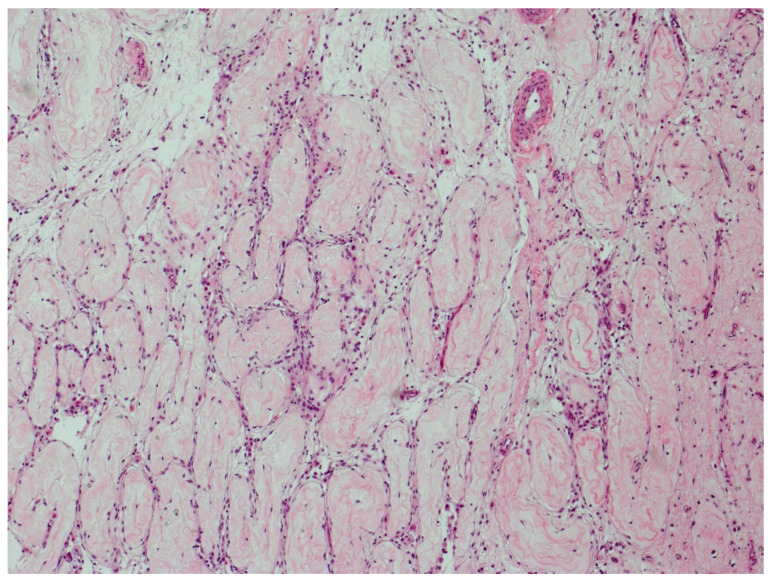
GCNIS cleared by host response. Parenchyma from which the host response has completely cleared GCNIS over time. All tubules have changed into thin fibrotic strands with scattered lymphocytes. (hematoxylin and eosin staining ×50 magnification).

Figure 3 illustrates parenchyma from which the host response has cleared GCNIS over time.

Lymphocytic infiltration increased with age among all three histological subgroups (Figure S3). The age pattern of tubules with infiltrating lymphocytes differed by histological subgroup. Whereas pure nonseminoma and seminoma-plus-nonseminoma showed an increase in tubules with infiltrating lymphocytes with age, their prevalence sharply decreased with age in pure seminoma (Figure S4).

Moderate or extensive lymphocytic infiltration was more frequently present in seminoma (66.2%) and seminoma-plus-nonseminoma (70.4%) compared with nonseminoma (38.9%). The prevalence of GCNIS was considerably lower among pure seminoma (59.9%) compared with pure nonseminoma (75.1%) and seminoma-plus-nonseminoma (89.2%). The prevalence of TM was higher in seminoma-plus-nonseminoma (37.8%) compared with seminoma (21.8%) and nonseminoma (25.4%) (Table 3). The prevalence of TM decreased with age among all histological subgroups, most clearly among pure seminomas (Figure S5).

The percentage of histological components such as seminoma, embryonal carcinoma, choriocarcinoma, yolk sac tumor, teratoma, and teratoma with somatic type malignancy was not associated with the presence of TM (data not shown). However, when the data were stratified by histological group, we found that TM occurred more frequently among familial nonseminoma (prevalence difference 10.7%, 95%CI 6.3; 23.1) and familial seminoma plus nonseminoma (prevalence difference 10.8%, 95%CI −11.1; 32.8) than among the corresponding sporadic tumors (Table S1).

TM and GCNIS were positively associated. In the absence of “some to many” GCNIS, the prevalence of TM was 15.9% whereas this prevalence was 30.1% in presence of GCNIS (prevalence difference +14.2%, 95%CI +6.7; +22.6). Lymphocytic infiltration was not associated with the presence of TM. GCNIS was not associated with lymphocytic infiltration (Tables S2 and S3).

## 4. Discussion

To the best of our knowledge, this is the only study comparing the histology of familial and sporadic cases of TGCT and, by combining the familial and sporadic cases, the largest study ever reported on the association between age at diagnosis of testicular TGCT and presence of GCNIS, TM, lymphocytic infiltration, and tubules with infiltrating lymphocytes. The examination of histology, histological components, lymphocytic infiltration, GCNIS and TM found no relevant differences between sporadic and familial TGCTs. A multivariable prediction model that simultaneously included all histological variables produced little discrimination beyond chance. The presence of GCNIS was negatively associated with age among pure seminoma and the presence of lymphocytic infiltration increased with age at diagnosis among all three histological subgroups. In contrast, the prevalence of TM decreased with age among all histological subgroups. Whereas pure nonseminoma and seminoma plus nonseminoma showed an increase in the prevalence of tubules with infiltrating lymphocytes with age, the prevalence sharply decreased with age among pure seminoma.

The multivariable logistic regression model that simultaneously used all histological characteristics we studied showed that sporadic and familial GCTs could not be distinguished by these characteristics. Thus, we did not confirm our hypothesis that specific histologic characteristics among familial cases might reflect etiologic heterogeneity which would permit more efficient susceptibility gene discovery. The results are consistent with the notion that the various variants of testicular type II GCTs share the same, essentially developmental, pathogenesis [10].

Testicular microlithiasis (TM) is characterized by calcifications in the seminiferous tubules and is considered a sign of dysgenesis of these tubules [23]. The prevalence of TM ranges from 2.4% to 5.6% among healthy adult men [24]. TM has been reported to be associated with Down syndrome, McCune–Albright syndrome, and the testicular dysgenesis syndrome including cryptorchidism, infertility, and also with a familial disposition of testicular cancer [25]. Tissue regions with clustering of TM may be positively associated with intratubular GCNIS [26]. In addition, a meta-analysis reported a 12-fold increased incidence of testicular cancer among subjects with TM [27]. 

Our estimated prevalence of TM of 25.3% is clearly higher than expected from surveys among healthy adult men (2.4–5.6%) [24] but remarkably lower than in other studies of TGCT. A small ultrasound-based study found that 22 out of 46 familial GCTs were positive for TM [12], however in contrast to ultrasound, which probes the whole testis, the current histological study examined only one slide per testis. Of note, the cases in the current analysis included a subset of cases from that published study.

Sharmeen et al. (2015) examined the prevalence of TM among TGCTs based on the review of pathology reports. The report found that while the histological percentage of seminoma was positively associated with TM, the histological percentage of embryonal carcinoma was negatively associated [28]. We were not able to corroborate these findings. Furthermore, Sharmeen et al. found that the TM count was associated with an earlier stage at diagnosis. However, among TGCTs composed of more than 50% embryonal carcinoma, there was no association with tumor stage. Unfortunately, we could not study this question as we had no data on stage at diagnosis. 

Pedersen et al. compared the prevalence of TM between Danish and British TGCTs based on histological slide review of eight slides on average. They found a prevalence of 19% (Denmark) and 41% (British). Although the number of slides was comparable between these two groups, different sampling techniques may have influenced this difference. As Pedersen et al. dichotomized age at diagnosis, interpretation of an age-related trend is not possible. Whereas the Danish tumors had a TM prevalence of TM of 20.2% for ages < 40 years and 17.2% ≥ 40 years, the corresponding percentages among the British tumors were 38.6% and 44.4% [29].

Using sonographic reports to examine TM among 123 TGCTs and 14 non-TGCT testicular tumors Heller et al. found a decreasing prevalence of TM with age at diagnosis (<30 years: 43.3%, 30–39 years: 38.8%, ≥40 years: 35.0%) [30]. To the best of our knowledge, we are the first to report a detailed histological analysis on age at diagnosis and prevalence of TM. In our case group of 533 TGCT cases, we found that per 5-year increase in the age at diagnosis, the odds of TM decreased by 17% (OR = 0.83, 95%CI 0.74–0.93).

The decline in TM prevalence by age suggests that TM may dissolve with age, which is conceivable in view of its ‘simple’ chemical composition of hydroxyapatite [31]. In crystal arthropathy, a self-limiting disease, hydroxyapatite is deposited in crystals, which may be surrounded by macrophages and foreign body-giants cells [32]. A reaction of this kind is lacking in testicular microlithiasis. This suggests that the age-related disappearance of microliths is a chemical process, not mediated by inflammatory cells. We found that the presence of occasional or many areas with GCNIS was associated with the presence of TM as has been previously described [33]. As such, it has been postulated that the presence of TM in association with GCNIS is an additional manifestation of the testicular dysgenesis syndrome [34].

The prevalence of TM among familial GCT cases depended on the presence of nonseminoma, which may explain some heterogeneity of the prevalence estimates in previous studies. Coffey et al. reported a higher prevalence of TM among familial TGCT cases (43.9%) compared with sporadic TGCT cases (34.4%) [35]. When we studied the association between TM and familial TGCT by histological group, we found that the prevalence of TM differs mainly between sporadic and familial nonseminoma.

In an analysis of 150 seminoma patients, Parker et al. found that amount of lymphocytic infiltration was higher among patients aged >33 years than younger patients [36]. We were able to corroborate their finding among seminoma patients, and additionally observed a similar positive association between age at diagnosis and amount of lymphocytic infiltration within the group of all remaining GCTs (nonseminoma and seminoma plus nonseminoma). On the available H and E-stained slides, the inflammatory infiltrates in seminomas and nonseminomas looked similar; however, they seemed to be directed towards different targets (see below). Parker et al. found that a higher amount of lymphocytic infiltration was associated with a better 10-year relapse-free survival [36]. We did not have follow up data, and therefore could not relate our data to survival. 

The prevalence of tubules with infiltrating lymphocytes increased by age for pure nonseminoma and seminoma-plus-nonseminoma whereas it decreased for pure seminoma. The increase for pure nonseminoma and seminoma-plus-nonseminoma suggests that this reaction gradually builds up over time. The decrease in the case of seminoma is probably due to the gradual disappearance of GCNIS, the presumed target of the host response. In each age group, the prevalence of tubules with infiltrating lymphocytes was higher in seminoma plus nonseminoma than pure nonseminoma, suggesting that the host response towards the tubules is stronger in seminoma than in nonseminoma.

The prevalence of GCNIS decreased for seminoma-plus-nonseminoma and pure seminoma with increasing age. However, among pure nonseminoma, there was barely any association with age. These findings confirm an earlier study [11] and are consistent with the hypothesis that the host reaction elicited by invasive seminoma, which may lead to a burnt-out seminoma, also affects GCNIS to the point that it, too, may be completely eradicated over time. The morphological similarity of the scar of a burnt-out seminoma with scattered lymphocytes and the entirely fibrotic tubules with sparse lymphocytes is indeed striking. The inflammatory response elicited by nonseminoma, also associated with lymphocytes infiltrating tubules, does not seem to affect GCNIS. This makes sense because the lymphocytic infiltration in nonseminomas does not target seminoma cells and should therefore not recognize GCNIS cells. These are novel observations which were possible because the morphological findings could be related to age of the patients in this large cohort.

There are several limitations that need to be borne in mind in interpreting these results. First, the blinded central pathology review of the tumors by two expert pathologists was based on a single HE-stained slide only. However, each reviewed slide was deemed to be representative of the overall case by the submitting pathologist. Second, the sampling procedure of sporadic cases does not guarantee that these cases are sporadic cases, since the family history was not available in the pathology archives with the exception of Norway. However, the probability that a randomly selected TGCT in the pathology archives is a familial case is very low, since only 1.4% of unselected TGCT report a positive family history [37]. Third, due to lack of clinical information in the pathology records, our findings could not be correlated with treatment and follow-up data.

## 5. Conclusions

In this study, we found no discernible systematic histological differences between familial and nonfamilial testicular germ cell tumors, and thus were unable to identify features that might indicate genetically distinct disease subsets. Our novel observations, including increasing lymphocytic infiltration, decreasing GCNIS and decreasing TM with increasing age at diagnosis, suggest a progressive host response that predominantly affects seminomas, the seminoma component of nonseminomas, and GCNIS, while TM may gradually dissolve with age.

## Figures and Tables

**Table 1:** Demographic characteristics and histological subtypes of 601 sporadic and familial testicular germ cell tumors.

Characteristic	Overall (*n* = 601)		Sporadic GCT (*n* = 296)		Familial GCT (*n* = 305)	
Center, N, %						
Norway	131	21.8	58	20.0	73	23.9
United Kingdom	324	53.9	156	52.7	168	55.1
United States	146	24.3	82	27.7	64	21.0
Age at diagnosis (years), N, %						
<20	36	6.0	19	6.4	17	5.6
20–24	83	13.8	44	14.9	39	12.8
25–29	128	21.3	56	18.9	72	23.6
30–34	123	20.5	57	19.3	66	21.6
35–39	85	14.1	42	14.2	43	14.1
40–44	61	10.2	32	10.8	29	9.5
45–49	33	5.5	17	5.7	16	5.3
50–54	31	5.2	18	6.1	13	4.3
55+	14	2.3	7	2.4	7	2.3
Missing	7	1.2	4	1.4	3	1.0
Mean age & SD	33.2	9.8	33.4	10.2	33.0	9.5
Age range	14–73		15–73		14–66	
Histological type, N, %						
Pure seminoma	299	49.8	135	45.6	164	53.8
Pure nonseminoma (≥1 N)	221	36.8	118	39.9	103	33.8
Seminoma + nonseminoma	81	13.5	43	14.5	38	12.5
Presence of histologic components %						
Seminoma, classical		62.2		58.8		65.6
Seminoma, HMI		0.5		0.7		0.3
Spermatocytic tumor		0.5		0.7		0.3
Embryonal carcinoma		40.6		42.6		38.7
Yolk sac tumor		33.0		34.1		31.8
Choriocarcinoma		2.8		3.0		2.6
Teratoma		29.6		32.1		27.2
Teratoma WMA		0.8		0.3		1.0

SD: standard deviation; pure nonseminoma (≥1 N): includes pure nonseminoma and mixed TGCTs with more than one nonseminoma component; HMI: high mitotic index; WMA: with malignant areas.

**Table 2:** Germ cell neoplasia in situ (GCNIS), testicular microlithiasis (TM), lymphocytic infiltration and tubules with infiltrating lymphocytes among sporadic and familial testicular germ cell tumors.

Characteristic	Overall		Sporadic GCT (*n* = 296)		Familial GCT (*n* = 305)		Prev. Difference	95%CI
	N	%	N	%	N	%		
GCNIS								
missing	81		37		44			
none	89	17.1	45	17.4	44	16.9		
occasional	69	13.3	38	14.7	31	11.9		
some	86	16.5	38	14.7	48	18.4		
many	276	53.1	138	53.3	138	52.9		
some-many	362	69.6	176	68.0	186	71.3	+3.3	−4.6; +11.2
TM								
missing	68		33		35			
no	398	74.7	202	76.8	196	72.6		
yes	135	25.3	61	23.2	74	27.4	+4.2	−3.2; +11.6
Lymphocytic infiltration								
none	21	3.5	9	3.0	12	3.9		
slight	239	39.8	119	40.2	120	39.3		
moderate	243	40.4	115	38.9	128	42.0		
extensive	98	16.3	53	17.9	45	14.8		
mod. + extensive	341	56.7	168	56.8	173	56.7	0.0	−8.0; +8.0
Tubules with infiltrating lymphocytes								
missing	86		37		49			
none	128	24.6	66	25.5	62	23.7		
occasional	138	26.5	72	27.8	66	25.2		
some	100	19.2	50	19.3	50	19.1		
many	149	28.6	71	27.4	78	29.8		
some-many	249	48.4	121	46.7	128	50.0	+3.3	−5.4; +11.9

Note: prevalence differences are presented with the reference group comprising the remaining categories.

**Table 3:** Lymphocytic infiltration, germ cell neoplasia in situ (GCNIS), testicular microlithiasis (TM), and tubules with infiltrating lymphocytes in relation to histological subtype.

	Pure Nonseminoma (≥1 N)	Mixed (Seminoma + Nonseminoma)	Pure Seminoma
Lymphocytic infiltration	*n* = 221	*n* = 81	*n* = 299
none %	5.9	0	2.7
slight %	55.2	29.6	31.1
moderate %	32.1	46.9	44.8
extensive %	6.8	23.5	21.4
moderate-extensive %	38.9	70.4	66.2
Prevalence difference (95%CI)	Ref.	+31.5 (+19.6; +43.3)	+27.3 (+18.9; +35.7)
GCNIS	*n* = 189	*n* = 74	*n* = 257
none %	12.2	6.8	23.7
occasional %	12.7	4.1	16.3
some %	15.3	13.5	18.3
many %	59.8	75.7	41.6
some-many %	75.1	89.2	59.9
Prevalence difference (95%CI)	Ref.	+14.1 (+4.7; +23.4)	−15.2 (−23.8; −6.6)
Microlithiasis	*n* = 193	*n* = 74	*n* = 266
Present %	25.4	37.8	21.8
Prevalence difference (95%CI)	Ref.	+12.5 (−0.2; +25.1)	−3.6 (−11.5; +4.3)
Tubules with infiltrating lymphocytes	*n* = 189	*n* = 74	*n* = 258
none	30.2	10.8	24.4
occasional	32.8	14.9	25.2
some	17.5	18.9	20.5
many	19.6	55.4	27.5
some-many	37.0	74.3	49.2
Prevalence difference (95%CI)	Ref.	+37.3 (+25.2; +49.4)	+12.2 (+2.9; +21.4)

*n*: number of cases in the data analysis; estimated prevalence differences with 95% confidence intervals in comparison to pure nonseminoma, ref.: reference group.

## Data Availability

The data presented in this study are available on request from the corresponding author.

## Supplementary Materials

The following are available online at https://www.mdpi.com/article/10.3390/cancers13071652/s1, Figure S1: Receiver operator curve and area under the curve for the association between histological variables and discrimination between sporadic and familial testicular germ cell tumors, Figure S2: Age dependence of germ cell neoplasia in situ (GCNIS) by histological groups (sporadic and familial TGCT combined), Figure S3: Age dependence of lymphocytic infiltration by histological groups (sporadic and familial TGCT combined), Figure S4: Age dependence of the prevalence of tubules with infiltrating lymphocytes by histological groups (sporadic ad familial TGCT combined), Figure S5: Age dependence of testicular microlithiasis (TM) by histological groups (sporadic and familial TGCT combined), Table S1: Germ cell neoplasia in situ (GCNIS), testicular microlithiasis (TM), lymphocytic infiltration, and tubules with infiltrating lymphocytes among sporadic and familial testicular germ cell tumors by histological subtype, Table S2: Association between testicular microlithiasis (TM) and germ cell neoplasia in situ (GCNIS), lymphocytic infiltration, and tubules with infiltrating lymphocytes, Table S3: Association between lymphocytic infiltration, germ cell neoplasia in situ (GCNIS), and tubules with infiltrating lymphocytes.
