# Supplementary material for: Histological Features of Sporadic and Familial Testicular Germ Cell Tumors Compared and Analysis of Age-Related Changes of Histology
Source: Cancers (Basel). 2021 Apr 1;13(7):1652. doi: 10.3390/cancers13071652 (PMC8037944; doi:10.3390/cancers13071652)
Supplement: Supplementary file 1 [file cancers-13-01652-s001.pdf]

cancers

MDPI

Supplementary Materials

Histological Features of Sporadic and Familial Testicular Germ Cell Tumors Compared and Analysis of Age-Related Changes of Histology

Andreas Stang, Mary L. McMaster, Isabell A. Sesterhenn, Elizabeth Rapley, Robert Huddart, Ketil Heimdal, Katherine A. McGlynn, J. Wolter Oosterhuis and Mark H. Greene

Digital Supplemental Material

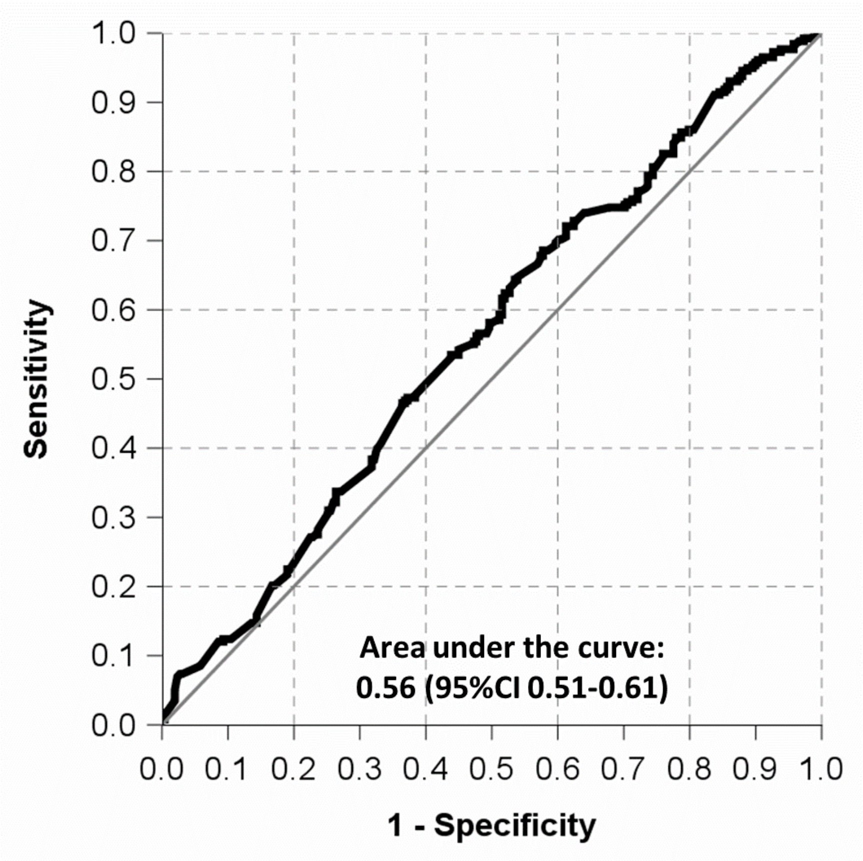

Figure S1. Receiver operator curve and area under the curve for the association between histological variables and discrimination between sporadic and familial testicular germ cell tumors. Model included amount of lymphocytic infiltration and germ cell neoplasia in situ (GCNIS), presence of testicular microlithiasis (TM), and percentage of each histological element; Hosmer-Lemeshow test (p = 0.33); 95%CI: 95% confidence interval.

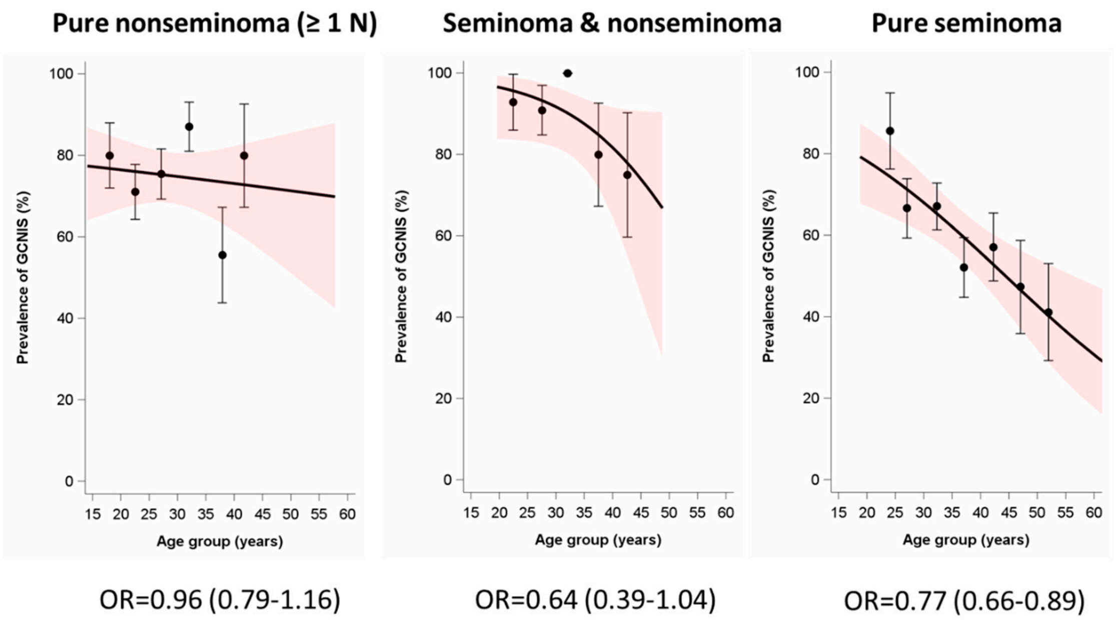

Figure S2. Age dependence of germ cell neoplasia in situ (GCNIS) by histological groups (sporadic and familial TGCT combined). Dots and whiskers indicate the age group specific prevalence +/− 1 standard error—when the prevalence was zero or one, only dots are plotted; only age groups with at least 10 subjects are plotted; the red bands display the 95% confidence interval bands; all models include age only; OR: odds ratio estimate per 5 year increment of age, in parenthesis 95% confidence intervals.

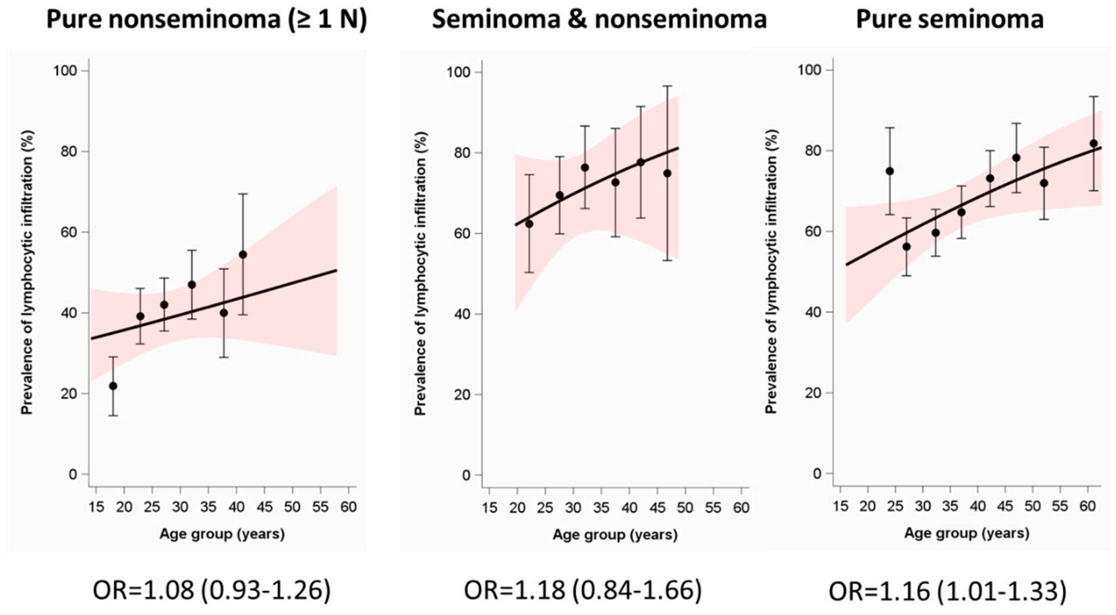

Figure S3. Age dependence of lymphocytic infiltration by histological groups (sporadic and familial TGCT combined). Dots and whiskers indicate the age group specific prevalence +/− 1 standard error—when the prevalence was zero or one, only dots are plotted; ; only age groups with at least 10 subjects are plotted; the red bands display the 95% confidence interval bands; all models include age only; OR: odds ratio estimate per 5 year increment of age, in parenthesis 95% confidence intervals.

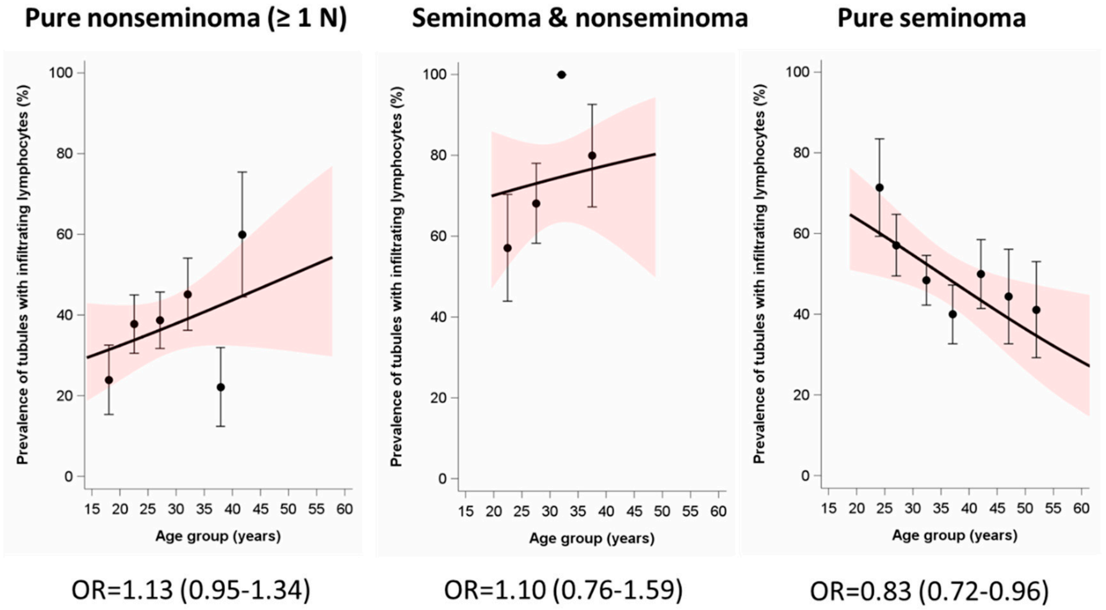

Figure S4. Age dependence of the prevalence of tubules with infiltrating lymphocytes by histological groups (sporadic and familial TGCT combined). Dots and whiskers indicate the age group specific prevalence +/− 1 standard error—when the prevalence was zero or one, only dots are plotted; only age groups with at least 10 subjects are plotted; the red bands display the 95% confidence interval bands; all models include age only; OR: odds ratio estimate per 5 year increment of age, in parenthesis 95% confidence intervals.

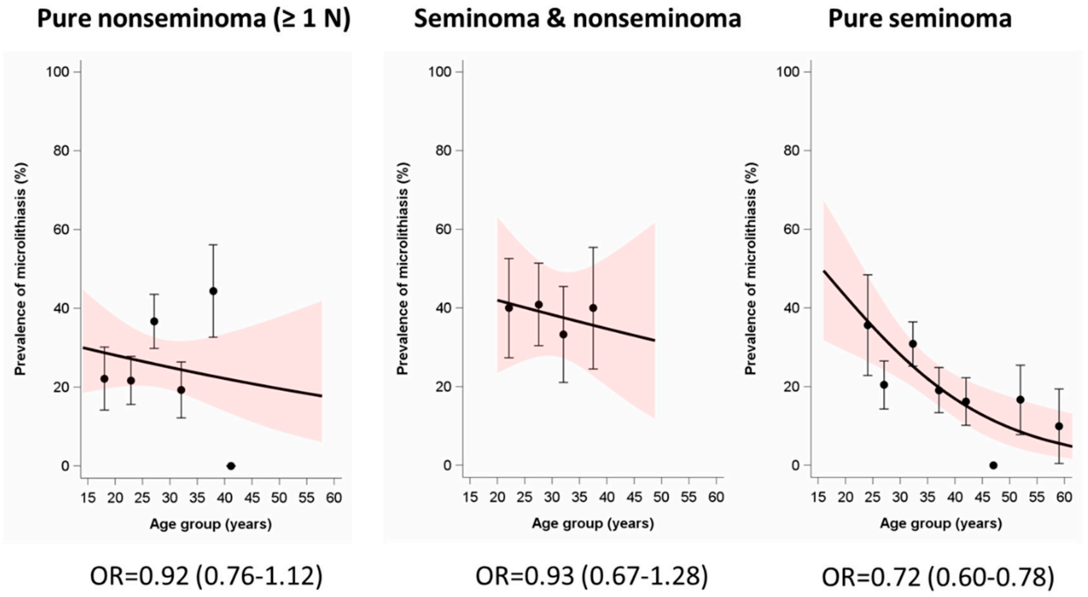

Figure S5. Age dependence of testicular microlithiasis (TM) by histological groups (sporadic and familial TGCT combined). Dots and whiskers indicate the age group specific prevalence +/− 1 standard error—when the prevalence was zero or one, only dots are plotted; only age groups with at least 10 subjects are plotted; the red bands display the 95% confidence interval bands; all models include age only; OR: odds ratio estimate per 5 year increment of age, in parenthesis 95% confidence intervals

Table S1. Germ cell neoplasia in situ (GCNIS), testicular microlithiasis (TM), lymphocytic infiltration, and tubules with infiltrating lymphocytes among sporadic and familial testicular germ cell tumors by histological subtype.

Characteristic	Overall		Sporadic GCT		Familial GCT		Prev. difference	95%CI
	N	%	N	%	N	%		
**Pure seminoma**								
GCNIS								
missing	42		15		27			
none-occas.	103	40.1	51	42.5	52	38.0		
some-many	154	59.9	69	57.5	85	62.0	+4.5	−16.6; +7.5
TM								
missing	33		12		21			
no	208	78.2	95	77.2	113	79.0		
yes	58	21.8	28	22.8	30	21.0	−1.8	−11.8; +8.2
Lymphocytic infiltration								
none-slight	101	33.8	44	32.6	57	34.8		
mod.-extens.	198	66.2	91	67.4	107	65.2	−2.2	−12.9; +8.9
Tubules with infiltrating lymphocytes								
missing	47		15		32			
none-occas.	128	50.8	64	53.3	64	48.5		
some-many	124	49.2	56	46.7	68	51.5	+4.9	−7.5; +17.2
**Pure nonseminoma (≥ 1 N)**								
GCNIS								
missing	32		16		16			
none-occas.	47	24.9	27	26.5	20	23.0		
some-many	142	75.1	75	73.5	67	77.0	+3.5	−8.8; +15.8
TM								
Missing	28		15		13			
No	144	74.6	82	79.6	62	68.9		
Yes	49	25.4	21	20.4	28	31.1	+10.7	+6.3; +23.1
Lymphocytic infiltration								
none-slight	135	61.1	71	60.2	64	62.1		
mod.-extens.	86	38.9	47	39.8	39	37.9	−2.0	−14.8; +10.9
Tubules with infiltrating lymphocytes								
missing	32		16		16			
none-occas.	119	63.0	62	60.8	57	65.5		
some-many	70	37.0	40	39.2	30	34.5	−4.7	−18.5; +9.0
**Seminoma plus nonseminoma**								
GCNIS								
missing	7		6		1			
none-occas.	8	10.8	5	13.5	3	8.1		
some-many	66	89.2	32	86.5	34	91.9	+5.4	−8.7; +19.5
TM								
Missing	7		6		1			
No	46	62.2	25	67.6	21	56.8		
Yes	28	37.8	12	32.4	16	43.2	+10.8	−11.1; +32.8
Lymphocytic infiltration								
none-slight	24	29.6	13	30.2	11	29.0		
mod.-extens.	57	70.4	30	69.8	27	71.0	+1.3	−18.6; +21.2
Tubules with infiltrating lymphocytes								
missing	7		6		1			
none-occas.	19	25.7	12	32.4	7	18.9		
some-many	55	74.3	25	67.6	30	81.1	+13.5	−6.2; +33.2

Pure nonseminoma (≥ 1N): includes pure nonseminoma and mixed TGCTs with more than one nonseminoma component.

Table S2. Association between testicular microlithiasis (TM) and germ cell neoplasia in situ (GCNIS), lymphocytic infiltration, and tubules with infiltrating lymphocytes.

Characteristic	TM Absent		TM Present		Prevalence Difference	95%CI
	N	%	N	%		
GCNIS						
none	72	81.8	16	18.2		
occasional	60	87.0	9	13.0		
some	70	82.4	15	17.7		
many	181	66.1	93	33.9		
some-many						
no	132	84.1	25	15.9	Ref.	
yes	251	69.9	108	30.1	+14.2	+6.7; +21.6
Lymphocytic infiltration						
none	13	76.5	4	23.5		
slight	154	75.9	49	24.1		
moderate	164	74.9	55	25.1		
extensive	67	71.3	27	28.7		
Lymphocytic infiltration, dichotomized						
none-slight	167	75.9	53	24.1	Ref.	
mod.-extensive	231	73.8	82	26.2	+2.1	−5.4; +9.6
Tubules with infiltrating lymphocytes						
none-occas.	213	80.7	51	19.3	Ref.	
some-many	167	67.6	80	32.4	+13.1	+5.5; +20.6

Table S3. Association between lymphocytic infiltration, germ cell neoplasia in situ (GCNIS), and tubules with infiltrating lymphocytes.

Characteristic	Lymphocytic Infiltration: Low		Lymphocytic Infiltration: High		Prevalence Difference	95%CI
	N	%	N	%		
GCNIS						
none	39	43.8	50	56.2		
occasional	31	44.9	38	55.1		
some	34	39.5	52	60.5		
many	109	39.5	167	60.5		
some-many						
no	70	44.3	88	55.7	Ref.	
yes	143	39.5	219	60.5	+4.8	−4.4; +14.0
Tubules with infiltrating lymphocytes						
none-occas.	137	51.5	129	48.5		
some-many	75	30.1	174	69.9	+21.4	+13.1; +29.7
